# Biomarkers of Oxidative Stress in Systemic Lupus Erythematosus Patients with Active Nephritis

**DOI:** 10.3390/antiox12081627

**Published:** 2023-08-17

**Authors:** Lu Liu, Karina de Leeuw, Suzanne Arends, Berber Doornbos-van der Meer, Marian L. C. Bulthuis, Harry van Goor, Johanna Westra

**Affiliations:** 1 Department of Rheumatology and Clinical Immunology, University Medical Centre Groningen, 9713 GZ Groningen, The Netherlands; l.liu01@umcg.nl (L.L.); k.de.leeuw@umcg.nl (K.d.L.); s.arends@umcg.nl (S.A.); b.doornbos-van.der.meer@umcg.nl (B.D.-v.d.M.); 2 Department of Pathology and Medical Biology, University Medical Centre Groningen, 9713 GZ Groningen, The Netherlands; m.bulthuis01@umcg.nl (M.L.C.B.); h.van.goor@umcg.nl (H.v.G.)

**Keywords:** reactive oxygen species (ROS), free thiols, malondialdehyde (MDA), soluble receptor for advanced glycation end products (sRAGE)

## Abstract

Oxidative stress plays an important role in systemic lupus erythematosus (SLE) and especially in lupus nephritis (LN). The aim of this study was to compare redox-related biomarkers between patients with active LN, quiescent SLE (Q-SLE) and healthy controls (HC) and to explore their association with clinical characteristics such as disease activity in patients. We investigated levels of plasma free thiols (R-SH, sulfhydryl groups), levels of soluble receptor for advanced glycation end products (sRAGE) and levels of malondialdehyde (MDA) in SLE patients with active LN (*n* = 23), patients with quiescent SLE (*n* = 47) and HC (*n* = 23). Data of LN patients who previously participated in Dutch lupus nephritis studies and longitudinal samples up to 36 months were analyzed. Thiol levels were lower in active LN at baseline and Q-SLE patients compared to HC. In generalized estimating equation (GEE) modelling, free thiol levels were negatively correlated with the Systemic Lupus Erythematosus Disease Activity Index (SLEDAI) over time (*p* < 0.001). sRAGE and MDA were positively correlated with the SLEDAI over time (*p* = 0.035 and *p* = 0.016, respectively). These results indicate that oxidative stress levels in LN patients are increased compared to HC and associated with SLE disease activity. Therefore, interventional therapy to restore redox homeostasis may be useful as an adjunctive therapy in the treatment of oxidative damage in SLE.

## 1. Introduction

Systemic lupus erythematosus (SLE) is a heterogeneous autoimmune disease associated with severe organ damage. The etiopathogenesis is a complex interplay between autoantibody production, defective clearance of apoptotic cells, chronic inflammation, loss of self-tolerance and abnormalities of the innate and adaptive immune system triggered by genes and environmental factors [1,2]. SLE is most common in women of childbearing age and of non-white ethnicity [3]. SLE is characterized by systemic symptoms and multi-organ involvement [3]. A large, wide range of autoantibodies are produced in SLE patients [4], some of which are involved in the metabolism of proteins, lipids and nucleotides, with association with disease activity.

Lupus nephritis (LN) is regarded as one of the most severe manifestations of SLE. The gold standard for diagnosis and classification of LN remains a kidney biopsy. Renal pathology is divided into six classes based on the location of immune complex deposits in the glomeruli, the extension of glomerular involvement and whether the type of injury is acute or chronic [5]. Classes III and IV are characterized by proliferative patterns and are considered to be the more active forms. They are treated with antimalarials and corticosteroids in combination with intensive immunosuppressive drugs (e.g., intravenous cyclophosphamide or mycophenolate mofetil) [6]. A novel modality for treating LN by reducing oxidative stress and thereby ameliorating immune cell dysfunction has been suggested [7]. Levels of anti-dsDNA titers, the amount of proteinuria and cells in urine are used to monitor treatment response and/or recurrence of LN.

Reactive oxygen species (ROS) are incomplete oxygen reduction byproducts produced by mitochondria or cytosolic enzymes during oxidative phosphorylation, which oxidize their surrounding molecules and have the ability to alter cellular metabolites, mediate cytotoxicity and regulate immune cell signaling [8]. ROS have been associated with various autoimmune diseases [9]. In SLE, chronic inflammation may lead to excessive production of ROS, inducing oxidative stress, which in turn causes DNA damage and cell necrosis of local epithelial and endothelial cells, thereby contributing to the release of self-reactive T and B cells that drive the amplification of this inflammatory response [10]. Indeed, it has been demonstrated that markers of oxidative stress correlate with disease activity [11]. The damaging effect of ROS is limited by antioxidant defense, which is particularly dependent upon synthesis of thiol proteins [12]. The kidney is a highly metabolic organ with abundant mitochondria, so aberrant or excessive redox reactions in the renal tissues of LN patients might be additionally harmful.

The exact pathophysiological role of oxidative stress in autoimmune diseases is not completely understood, although some progress has been achieved in investigating oxidative stress markers. An increasing number of studies are shifting their focus to investigating oxidative stress biomarkers as a readout for possible additional treatment with antioxidant drugs to prevent this redox disequilibrium [13].

Several biomarkers of oxidative stress are known. For example, plasma free thiols (R-SH, sulfhydryl groups) are compounds that are common targets of the Reactive Species Interactome (RSI) [14]. Free thiols reliably reflect systemic oxidative stress since they are readily oxidized by reactive species [15,16]. Blood proteins, mainly albumin, harbor the largest amount of redox-active thiol groups (approximately 75% of the total thiol pool). Another possible biomarker is the soluble receptor for advanced glycation end products (sRAGE). The canonical ligands of the RAGE are advanced glycation end products (AGEs), which are produced as a consequence of excessive ROS-induced cell and organ damage. sRAGE acts as a decoy receptor for AGEs, dampening the pro-inflammatory effects of AGEs in SLE [17], so levels of sRAGE may represent oxidative stress. ROS production is also associated with lipid peroxidation, which can be measured by malondialdehyde (MDA), a strong reactive aldehyde [18,19]. Excessive ROS produced in SLE patients interact with the lipid membranes of cells, resulting in a notable elevation in MDA levels [12].

The aim of this study was to compare levels of thiols, sRAGE and MDA between patients with active LN, quiescent SLE and HC. Furthermore, associations between these oxidative stress biomarkers and parameters of disease activity will be investigated to elucidate whether these biomarkers can be used to monitor disease activity and might be targets for future additional treatment modalities.

## 2. Materials and Methods

### 2.1. Patient Selection

Plasma samples of a previously conducted lupus nephritis (LN) study were used in this study [20,21]. In this study, 87 patients with systemic lupus erythematosus (SLE) with proliferative LN were randomly assigned to a 2 year treatment with either azathioprine (AZA, 2 mg/kg/day) combined with intravenous methylprednisolone (3 × 3 pulses of 1000 mg) and oral prednisone (initially 20 mg/day) or intravenous cyclophosphamide (ivCY, 750 mg/m^2^, 13 pulses in 2 years) combined with oral prednisone (initially 1 mg/kg/day). Twenty-three patients of this study were included between in the present study based on availability of plasma samples, and the moment of inclusion was called baseline or 0 months. All patients were aged between 18 and 60 years and fulfilled ≥4 American College of Rheumatology (ACR) criteria for SLE; they had creatinine clearance >25 mL/min (Cockcroft–Gault formula) and biopsy-proven proliferative LN (WHO class III, IV, V c or V d). Inclusion and exclusion criteria have been described previously [22]. The study was approved by the ethics committee of all participating hospitals, and all patients provided written informed consent.

Patients were evaluated every six months, and during each visit, the SLE Disease Activity Index (SLEDAI) and other clinic parameters were measured, such as anti-double stranded DNA (anti-dsDNA), creatinine and proteinuria [23]. Furthermore, during follow-up, renal relapses were identified, measured as a doubling of the lowest obtained serum creatinine so far and/or proteinuric flare, or the development of a nephrotic syndrome (proteinuria > 3.5 g/day) in a previously non-proteinuric patient whose lowest protein excretion had repeatedly been 2.0 g/day or proteinuria > 1.5 g/day without other causes. In addition, 47 clinically quiescent SLE patients (SLEDAI score ≤ 4) fulfilling the ACR criteria or SLE International Collaborating Clinics criteria (SLICC), without severe organ involvement, excluding other connective tissue disease, all with a disease duration of <10 years and 23 healthy individuals matched for age and gender to the SLE patients were recruited as control group [24].

### 2.2. Measurements of Thiols, sRAGE and MDA

Levels of thiols (µmol/L) were measured in the same manner as previously described [25,26], with minor modifications. Briefly, seventy-five microliters of plasma samples was diluted 1:4 with 0.1 M Tris buffer (pH 8.2) and transferred to microplates. A Sunrise microplate reader (Tecan Trading AG, Männedorf, Switzerland) with a reference filter at 630 nm was used to measure background absorption at 412 nm. Subsequently, ten microliters of 3.8 mM 5,5-Dithio-bis (2-nitrobenzoic acid) (DTNB, CAS-number 69-78-3, Sigma Aldrich Corporation, Saint Louis, MO, USA) in a 0.1 M phosphate buffer (pH 7) was added to the samples. The absorption was measured again after 20 min of incubation at room temperature. The concentration of free thiols in the samples was compared with the absorbance of l-cysteine (CAS No. 52-90-4, Fluka Biochemika, Buchs, Switzerland) standards in 0.1 M Tris and 10 mM EDTA (pH 8.2) in a concentration range of 15.6–1000 M.

Levels of sRAGE were measured by an enzyme linked immunosorbent assay (ELISA) using sandwich ELISAs; the results were analyzed using a VersaMax microplate reader at 450–575 nm and sRAGE results were expressed in pg/mL. Levels of MDA (μmol/L) were measured using a commercial colorimetric tests Lipid Peroxidation Assay (MDA) kit (ab118970) according to manufacturers’ instructions; the results were read on a microplate reader at OD 532 nm.

### 2.3. Statistical Analysis

The results were expressed as number of patients (%) for categorical data and median (interquartile range; IQR) for non-normally distributed continuous data. Differences between the three groups in characteristics and biomarkers were analyzed using Chi-Square test followed by Chi-Square tests or a Kruskal–Wallis test followed by Mann–Whitney U tests, respectively. Spearman correlation coefficients were used to analyze the association between the redox-related biomarkers in patients with active LN, quiescent SLE and HC. Generalized estimating equations (GEE) with an exchangeable correlation structure were used to analyze the association of biomarkers of oxidative stress with clinical and laboratory parameters within LN patients over time [27]. If residuals were non-normally distributed, variables were transformed (log, square root) prior to being entered into the equation [28]. *p* values < 0.05 were considered statistically significant. The statistical analysis was performed with IBM SPSS Statistics 28.

## 3. Results

### 3.1. Cohort Demographics and Characteristics

Demographic characteristics are shown in Table 1. The median age of SLE patients with active LN was 34 years (IQR: 28.0–49.0) and 78% of patients were female. The median age of quiescent SLE patients was 43 years and 79% of patients were female. All LN patients were included during active disease, reflected by a median SLEDAI of 14, and all SLE patients had quiescent state at baseline, reflected by a median SLEDAI of 2. Levels of creatinine, thrombocytes and leukocytes were significantly higher in the LN group compared to quiescent SLE and HC groups. Levels of C3 were significantly lower and SLEDAI and utilization rates of prednisone and azathioprine were significantly higher in LN compared to quiescent SLE.

### 3.2. Levels of Biomarkers and Correlation of Biomarkers in Groups at Baseline

**Figure 1:**
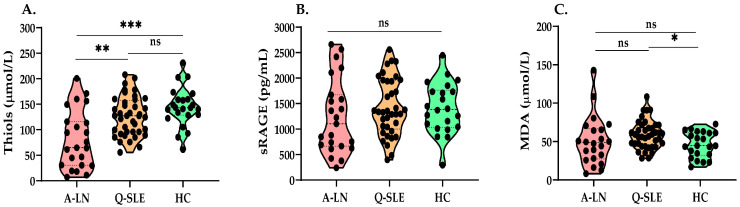
Levels of thiols, sRAGE and MDA at baseline in active LN, quiescent SLE and HC. Violin plots showing levels at baseline: (**A**) plasma free thiol levels, (**B**) sRAGE levels, (**C**) MDA levels. Abbreviations: A-LN: active lupus nephritis, Q-SLE: quiescent systemic lupus erythematosus, HC: healthy controls, sRAGE: soluble receptor for advanced glycation end products, MDA: malondialdehyde, ns: not significant, statistical significance: * *p* < 0.05, ** *p* < 0.002, *** *p* < 0.0002.

**Figure 2:**
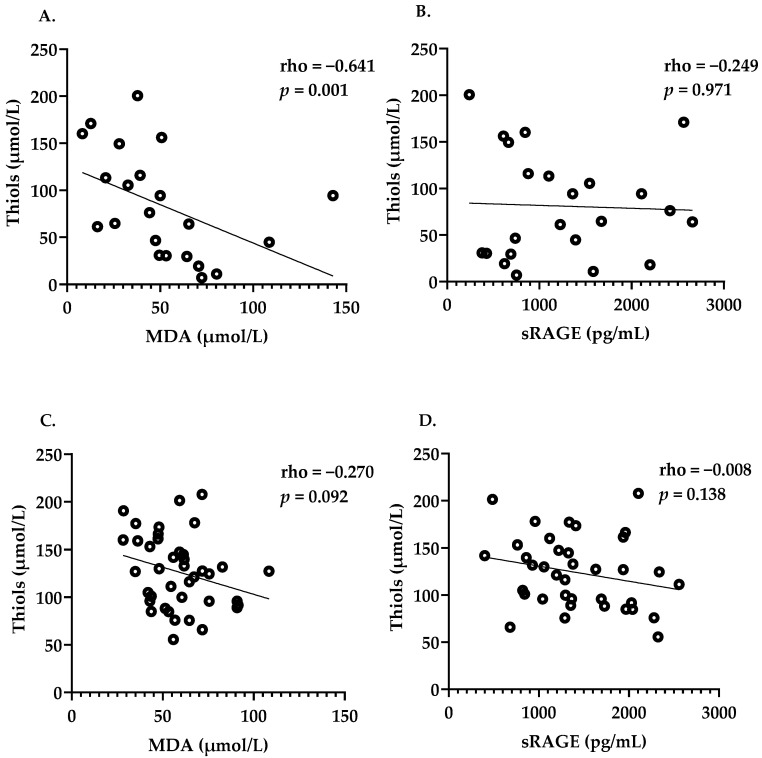

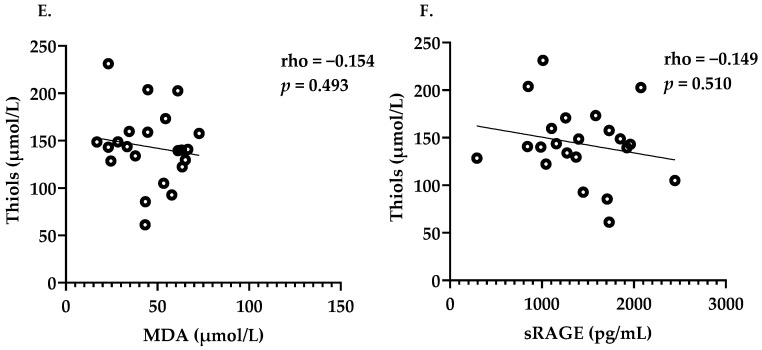
Correlation between oxidative stress biomarkers in active LN patients (**A**,**B**), quiescent SLE patients (**C**,**D**) and HC (**E**,**F**). rho: correlation coefficient; Spearman’s rank correlation was used. *p* < 0.05: statistical significance.

At baseline, plasma thiol levels in SLE patients with active LN were significantly decreased and lowered in quiescent SLE patients compared to HC (Figure 1A). sRAGE levels were not different between the groups, while MDA levels in quiescent SLE patients were significantly elevated compared to HC (Figure 1B,C). In patients with active LN, a significant negative correlation was found between MDA and levels of free thiols (rho = −0.64, Figure 2A), but not between thiols and sRAGE (Figure 2B). No significant correlations were seen between thiols and MDA and sRAGE in quiescent SLE (Figure 2C,D) and HC (Figure 2E,F). Additionally, no correlations were found among levels of oxidative markers with other disease-related markers at baseline.

### 3.3. LN Cohort in Longitudinal Study

**Figure 3:**
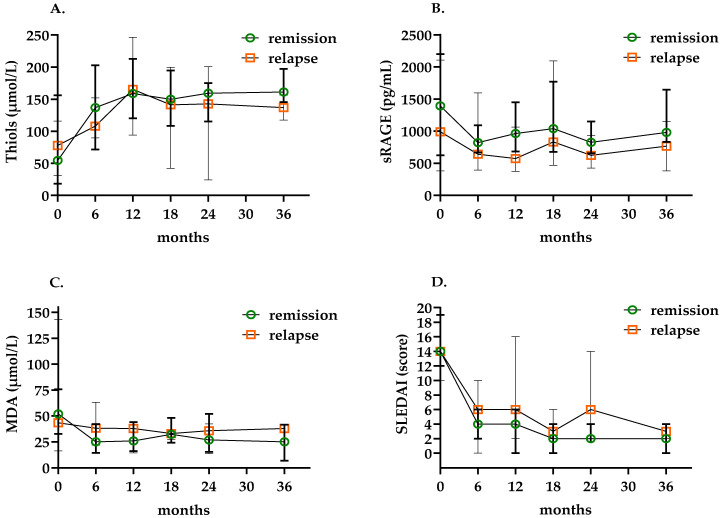
Median levels of oxidative stress biomarkers and SLEDAI scores during 36 months follow-up of LN patients: (**A**) median plasma-free thiol levels, (**B**) median sRAGE levels, (**C**) median MDA levels, (**D**) median SLEDAI levels. Green dots represent remission group, red dots represent relapse group; each dot with vertical lines indicates median with error. Abbreviations: SLEDAI: Systemic Lupus Erythematosus Disease Activity Index.

Next, we measured changes over time in oxidative biomarkers and SLEDAI in patients with LN during a follow-up of 36 months (Figure 3). Changes over time per individual patient are shown in Supplementary Figure S1. During follow-up, six patients experienced a flare-up, resulting in a recurrence rate of 26%. Remaining patients in remission showed a decrease in SLEDAI over time, while thiol levels increased (Figure 3A,D). Levels of sRAGE and MDA showed a slight decline over time (Figure 3B,C). In Figure 3, no significant differences were found between relapse and remission groups up to 24 months of follow up.

Finally, we used a GEE analysis to estimate the association between biomarkers of oxidative stress and clinical parameters over time (Table 2). Higher levels of free thiols were significantly associated with lower SLEDAI (B = −4.72, *p* < 0.001). Higher levels of sRAGE (sRAGE (B = 20.69, *p* = 0.035)) and higher levels of MDA (B = 0.84, *p* = 0.016) were significantly associated with higher SLEDAI.

We also observed that levels of free thiols were positively correlated with creatinine levels (B = 0.17, *p* = 0.048), and levels of MDA were negatively correlated with creatinine (B = −0.10, *p* = 0.048) and levels of leukocytes (B = −1.71, *p* = 0.044). No other significant correlations were found.

## 4. Discussion

In this study, we demonstrated that levels of plasma free thiols are decreased in SLE and LN patients compared to HC, while levels of sRAGE are comparable between the groups and levels of MDA are increased in Q-SLE, but not in LN compared to HC. However, all three redox-related biomarkers were associated with SLEDAI in a longitudinal analysis of up to 3 years in LN patients. This indicates that oxidative stress plays an important role in SLE and LN and that it might be a target of additional and new treatments.

The pathogenesis of LN is thought to be accelerated by an inflammatory loop of autoimmune reactions and oxidative stress [29]. Although moderate levels of ROS are necessary for physiological cell functions, excessive levels can induce cell and tissue damage [30]. Measuring oxidative stress is difficult; however, levels of plasma free thiols might be a relatively easy way to obtain a good impression. Thiols readily react with oxygen radicals to form disulfides and thus reflect the systemic status of the redox balance in the body. In addition, thiols reflect DNA repair capabilities and the possible eventual accumulation of genetic damage because the essential DNA repair enzyme, poly ADP-ribose polymerase (PARP), is regulated by thiols, with the added benefit that thiol levels are unaffected by chemotherapy and immunotherapy, which make them strong candidates for biomarkers [31]. Thiols, as oxidative stress markers, are used not only in reflecting DNA oxidization but are also employed in disease- and organ-specific investigations. A study of ischemia reperfusion injuries in renal transplants showed a lower oxidative stress was associated with a better early renal graft function [32]. A study conducted by Ates et al. in type 1 diabetes mellitus patients found that oxidative stress was positively correlated with glycated hemoglobin A1c levels [33]. However, this was not confirmed in another cohort [34]. A prospective study on fetal growth and preeclampsia revealed that levels of free thiols were decreased in growth-restricted fetuses and patients with preeclampsia [35]. The role of thiols in lupus has also attracted wider attention. Some studies confirmed our findings that thiol levels were negatively correlated with SLE disease activity and major organ involvement [36]. Other studies have elucidated that oxidative stress was even increased in classes III/IV LN compared to other classes. In addition, thiols can rapidly reflect changes in oxidative stress independent of immunosuppressive agents [37,38]. We found that thiol levels are increased in SLE patients with and without LN and are negatively correlated with disease activity, confirming a study which demonstrated increased oxidative stress in patients with active class III/IV lupus nephritis [38].

One ligand of RAGE is High Mobility Group Box 1 (HMGB1), and it has been reported that anti-DNA antibodies bound to HMGB1 show a synergistic pro-inflammatory effect on mesangial cells of LN in a RAGE-dependent manner. sRAGE, which is a soluble form of RAGE, can act as a decoy receptor and block RAGE signaling transduction [39,40]. Studies demonstrated a close bond between the production of AGEs, which act as the other ligands of sRAGE, and oxidative stress as well as hyperglycemia [41]. Hence, sRAGE might serve as an oxidative biomarker for monitoring changes in carbohydrate oxidation levels [42]. The role of sRAGE as a diagnostic marker of disease activity is controversial, because its level may increase in several diseases (autoimmune diseases, tumors, diabetes, etc.), but the results are still inconclusive [43]. Interestingly, it has been found that treated or untreated SLE patients have similar levels of sRAGE, although patients receiving long-term treatment have higher levels of sRAGE than patients receiving short-term treatment [44]. SLE patients with antiphospholipid antibodies or antiphospholipid syndrome also have lower sRAGE levels, possibly due to overconsumption of decoy receptors with increasing inflammation [45]. At last, decreased serum levels of sRAGE and a positive correlation with disease activity have also been found in SLE patients [44,46]. However, in our study, there was no difference between LN, SLE and HC levels. This might be explained by the fact that when patients receive long-term treatment, sRAGE levels gradually revert to normal levels, which indicates that either sRAGE may play different roles in the initial and progressing stage of the disease or a compensatory mechanism related to sRAGE production and regulation may be triggered during treatment.

Circulating MDA proteins are increased in autoimmune diseases and attributed to inflammation-altered regulation of oxidation [18]. A previous study indicated that increased oxidative stress and elevated MDA levels were positively associated with SLE disease activity [47]. In our study, we observed that MDA levels were significantly higher in quiescent SLE patients compared to HC. This finding is consistent with the results reported by Lalwani et al. [37]. Currently, changes in MDA levels in lupus patients are not entirely consistent. It is possible that a redox imbalance interacts with lipid peroxidation, a process that impacts glomerular basement membrane integrity and renal tubular functions [42]. Interestingly, we found that levels of MDA did not differ in active LN compared with HC.

Most studies on oxidative stress biomarkers in SLE have a cross-sectional design. In this follow-up cohort study, we could monitor changes in these biomarkers over time. In the GEE analysis, we observed statistically significant correlations between thiols and creatinine, as well as between MDA, creatinine and leukocytes. However, further validation of these findings is warranted.

Corticosteroids are crucial agents used to reduce inflammation and for immunosuppression and are considered as one of the most commonly used treatments in lupus; however, they also have a variety of adverse effects [48]. Oxidative stress potentially reduces the response to corticosteroids by affecting glucocorticoid receptor expression and signaling, which may lead to glucocorticoid resistance [49]. However, a study showed that there is no significant association between the use of corticosteroids and the production of ROS in polymorphonuclear leukocytes from SLE patients [50]. In our study, we did not see a difference in thiol levels in SLE patients who did or did not use steroids.

In a recent review, the effects of oxidative stress in SLE were described [51]. Additionally, several pharmacological approaches targeting oxidative stress are mentioned, such as rapamycin, which targets the mammalian target of rapamycin (mTor) pathway, but also other antioxidant molecules such as CoQ10. Recently, more attention has been given to the transcription factor NF-E2-related factor 2 (Nrf2) as a central regulator of cellular antioxidative responses, inflammation and restoration of the redox balance [52]. Targeting Nrf2 for the treatment of diseases associated with oxidative stress and inflammation, such as SLE, might be promising.

There are some limitations. The study was conducted in the Netherlands, with a small number of subjects, most of whom were Caucasian, and the generalizability of the results to other ethnicities is therefore unknown. Another limitation is that in our longitudinal cohort study, there was a smaller number of patients with a relapse; additionally, there were insufficient and misrepresentative data at some time points, which made it difficult to conduct statistical analyses to evaluate intergroup differences. Furthermore, this study only focused on proliferative LN patients. In the future, an evaluation of oxidative stress may be conducted in patients with other classifications. In addition, LN patients were included based on the availability of plasma samples, and we could not include the whole cohort.

## 5. Conclusions

In conclusion, these results suggest that SLE patients with or without renal involvement have increased oxidative stress levels compared with HC, and markers of oxidative stress are likely to be associated with disease activity. Levels of free thiols might be a better biomarker in LN compared to sRAGE and MDA. Moreover, adding antioxidant agents to the current treatment strategies of SLE might have a promoting effect by restoring the redox balance and alleviating various complications induced by oxidative stress.

## Figures and Tables

**Table 1:** Baseline characteristics of patients and healthy controls.

Characteristics	LN (*n* = 23)	SLE (*n* = 47)	HC (*n* = 23)	p1	p2
Age (years)	34 (28–49)	43 (29–54)	47 (28–61)	0.324	N.A.
Gender females, n (%)	18 (78%)	37 (79%)	18 (82%)	0.947	N.A.
Systolic blood pressure (mmHg)	120 (110–130)	120 (110–130)	120 (110–120)	0.77	N.A.
Diastolic blood pressure (mmHg)	80 (70–80)	75 (70–80)	75 (70–80)	0.867	N.A.
Weight (Kg)	67 (63–86)	72 (60–85)	69 (62–80)	0.859	N.A.
Thrombocytes (10^9/L)	303 (263–341)	230 (198–281)	236 (212–273)	0.006	AB = 0.007 AC = 0.036
Creatinine (umol/L)	81 (76–92)	72 (62–81)	71 (62–76)	<0.001	AB = 0.002 AC = 0.001
ALAT (U/L)	19 (12–29)	19 (15–23)	19 (14–28)	0.952	N.A.
Hemoglobin (mmol/L)	8.1 (7.4–8.3)	8.0 (7.7–8.5)	8.3 (8.0–8.9)	0.068	N.A.
Leukocytes (10^9/L)	9.2 (7.6–12.4)	5.4 (4.4–7.1)	5.6 (4.8–6.2)	<0.001	AB < 0.001 AC < 0.001
Complement 3 (g/L)	0.79 (0.7–1.0)	1.0 (0.8–1.1)	1.06 (0.9–1.2)	0.013	AB = 0.044 AC = 0.018
Complement 4 (g/L)	0.18 (0.1–0.3)	2.0 (2.0–4.0)	0.19 (0.2–0.3)	0.257	N.A.
SLEDAI, score	14 (12–19)	2 (2–4)	N.A.	N.A.	<0.001
Anti-dsDNA positive, n (%)	14 (61%)	21(45%)	N.A.	N.A.	0.203
Prednisone use, n (%)	23 (100%)	13 (28%)	N.A.	N.A.	<0.001
Azathioprine use, n (%)	13 (57%)	6 (13%)	N.A.	N.A.	<0.001

Values are number (percentage) or median (interquartile range), *p* < 0.05: statistical significance, p1: comparison of all groups, p2: pairwise comparison, A: LN, B: SLE, C: HC. Abbreviations: LN: lupus nephritis, SLE: systemic lupus erythematosus, HC: healthy controls, SLEDAI: Systemic Lupus Erythematosus Disease Activity Index, anti-dsDNA: anti-double-stranded DNA, ALAT: alanine aminotransferase, N.A.: not applicable.

**Table 2:** Association between oxidative stress biomarkers clinical and laboratory parameters in patients over time.

	Thiols				sRAGE				MDA			
	B	95% Confidence Interval		*p*-Value	B	95% Confidence Interval		*p*-Value	B	95% Confidence Interval		*p*-Value
SLEDAI	−4.72	−5.98	−3.46	<0.001 *	20.69	1.47	39.91	0.035 *	0.84	0.15	1.52	0.016 *
Hemoglobin	−0.23	−11.64	11.17	0.968	−54.45	−207.14	98.24	0.485	2.19	−4.03	8.41	0.490
Leukocytes	−0.81	−5.20	3.58	0.718	−5.58	−32.20	20.99	0.680	−1.71	−3.36	0	0.044 *
Thrombocytes	−0.10	−0.23	0.02	0.113	0.09	−1.74	1.92	0.923	0	−0.05	0.05	0.902
Complement 3	−17.06	−72.92	38.80	0.550	256.06	−258.55	770.68	0.329	16.64	−24.49	57.78	0.428
Complement 4	−130.99	−281.09	19.12	0.087	1490.08	−334.02	3314.19	0.109	40.20	−62.58	142.98	0.443
Creatinine	0.17	0	0.33	0.048 *	0.46	−2.42	3.33	0.756	−0.10	−0.21	0	0.048 *
urine protein/24 h	−1.28	−4.84	2.28	0.481	−5.21	−46.52	36.11	0.805	0.26	−1.10	1.61	0.713
Anti-dsDNA (pos vs. neg)	−12.64	−40.14	14.85	0.367	−184.13	−459.76	91.50	0.190	−6.63	−18.24	4.97	0.263

* p < 0.05—statistical significance.

## Data Availability

All data are contained within the present article.

## Supplementary Materials

The following supporting information can be downloaded at: https://www.mdpi.com/article/10.3390/antiox12081627/s1, Figure S1: Oxidative stress biomarkers and SLEDAI levels during 36 months follow-up of LN per individual patient. Spaghetti plot showing markers of oxidative stress levels (a.) plasma-free thiol levels, (b.) sRAGE levels, (c.) MDA levels, (d.) SLEDAI levels during 36 months follow-up in LN patients. Green lines represent remission group and red lines represent relapse group. sRAGE: soluble receptor for advanced glycation end products, MDA: malondialdehyde, SLEDAI: Systemic Lupus Erythematosus Disease Activity Index.
