# Supplementary material for: Biomarkers of Oxidative Stress in Systemic Lupus Erythematosus Patients with Active Nephritis
Source: Antioxidants (Basel). 2023 Aug 17;12(8):1627. doi: 10.3390/antiox12081627 (PMC10451241; doi:10.3390/antiox12081627)
Supplement: Supplementary file 1 [file antioxidants-12-01627-s001.zip › antioxidants-2542714-supplementary.pdf]

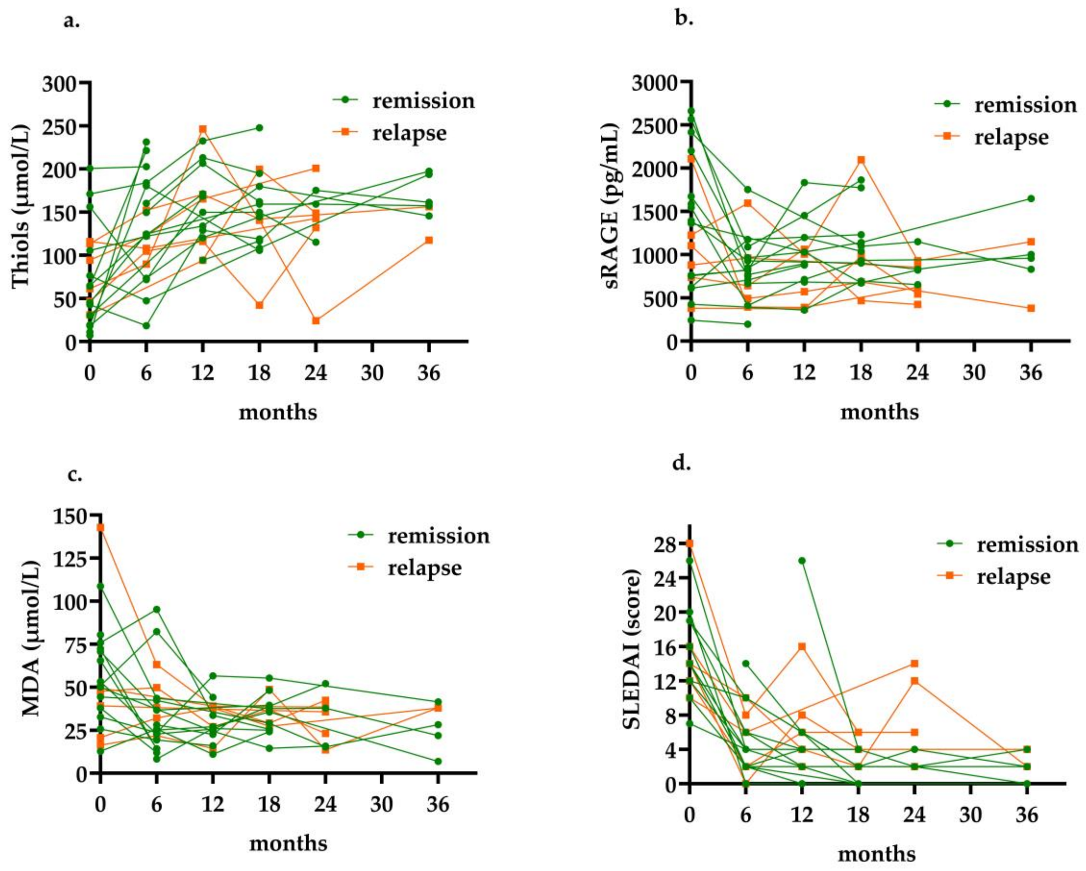

Figure S1: Oxidative stress biomarkers and SLEDAI levels during 36 months follow-up of LN per individual patient. Spaghetti plot showing markers of oxidative stress levels (a.) plasma-free thiol levels, (b.) sRAGE levels, (c.) MDA levels, (d.) SLEDAI levels during 36 months follow-up in LN patients. Green lines represent remission group and red lines represent relapse group. sRAGE: soluble receptor for advanced glycation end products, MDA: malondialdehyde, SLEDAI: Systemic Lupus Erythematosus Disease Activity Index.
